# Antioxidant and hepatoprotective effects of the methanol extract of the leaves of *Satureja macrostema*

**DOI:** 10.4103/0973-1296.62901

**Published:** 2010-05-05

**Authors:** Rosa Martha Perez Gutierrez, Yoja Teresa Gallardo Navarro

**Affiliations:** *Laboratorio de Investigación de Productos Naturales. Escuela Superior de Ingeniería Química e Industrias extractivas IPN. Punto fijo 16, Col. Torres Lindavista cp 07708. D.F. México*; 1 *Laboratorio de Tecnología de Alimentos, Carpio/Plan de Ayala S/N, Casco de Santo Tomas México D.F*.

**Keywords:** *Satureja Macrostema*, hepatoprotective activity, antioxidant effect, biochemical parameters

## Abstract

*Satureja Macrostema* is used both as a functional food and as a drug. In this study, the antioxidative potential of the methanol extract of *Satureja Macrostema* (SM) was evaluated using various antioxidant assays, including DPPH, superoxide, nitric oxide (NO), hydroxyl radical scavenging and iron-chelating activity. Total phenolic and flavonoid content of SM was also determined by a colorimetric method. The extract exhibited powerful free radical scavenging, especially against DPPH, hydroxyl radical scavenging and iron-chelating activity as well as a moderate effect on NO and superoxide anions. The protective effects of methanol extract of SM were studied in carbon tetrachloride-reduced biochemical markers of hepatic injury such as glutamate pyruvate transaminase (SGPT), serum glutamate oxalaoacetate transaminase (SGOT), alkaline phosphatase (ALP), serum bilirubin, cholesterol alanine aminotransferase (ALT) and aspartate aminotransferase (AST) levels. The increased level of HDL demonstrated dose dependant reduction in the *in vivo* peroxidation induced by CCl4. SM could protect from paracetamol-induced lipid peroxidation eliminating the deleterious effects of toxic metabolites from paracetamol. Degree of protection was measured by using biochemical parameters such as serum transaminase (GOT and GPT), alkaline phosphatase (ALKP) and bilirubin. Hexane and chloroform extracts did not show any effects. Results obtained in the present study suggest that S. Macrostema elicits hepatoprotectivity through antioxidant activity on carbon tetrachloride- and paracetamol-induced hepatic damage in rats.

## INTRODUCTION

The role of free radical reactions in disease pathology is well established, suggesting that these reactions are necessary for normal metabolism but can be detrimental to health; the antioxidants protected against free radicals induced oxidative damage by antioxidant enzymes such as superoxide dismutase and catalase or antioxidant compounds.[1] The liver is expected not only to perform physiological functions but also to protect against the hazards of harmful drugs and chemicals. Inspite of tremendous scientific advancement in the field of hepatology in recent years. Jaundice and hepatitis are two major hepatic disorders that account for a high death rate.[2] Liver diseases are mainly caused by toxic chemicals, excess consumption of alcohol, infections and autoimmune disorders. Most of the hepatotoxic chemicals damage liver cells mainly by inducing lipid peroxidation and other oxidative damages.[3] In spite of tremendous advances in modern medicine, there are no effective and reliable drugs available that can stimulate liver function, offer protection to the liver from damage or help to regenerate hepatic cells.[4] However, there are a number of medicinal preparations in Ayurveda that are recommended for the treatment of liver disorders.[5]

*Satureja Macrostema* (Moc. andand Sesse ex Benth.) belongs to the family Lamiaceae commonly known as “nurhiteni”. The aerial parts of the plant are used as spices in traditional Mexican cuisine. It is used for giving herbal bath to woman after child birth. Drinking of tea made from this herb thrice a day reduces stomach pain, soothes inflammation of the ovaries, improves gastrointestinal conditions, soothes gastric pains, stimulates bowel movements and helps in slowing down digestion. It is also used as an aphrodisiac in the treatment of bile and liver stones.[6] A survey of literature revealed that the pharmacological properties of this plant have not been scientifically investigated. The current investigation is an attempt to study the hepatoprotective and antioxidant activity of the hexane, chloroform and methanol extracts of *S. Macrostema*.

## MATERIALS AND METHODS

### Plant material

Fresh plants of *S. Macrostema* were collected from Oaxaca State, Mexico in December 2007. A voucher specimen was deposited in the Herbarium of the CIDIR-Oaxaca for further reference (No.6456).

### Animals

The study was conducted in male Wistar strain albino rats, weighing about 180–225 g. They were housed in microlon boxes in a controlled environment (temperature 25 ± 2 °C) with standard laboratory diet and water *ad libitum*. The animals were acclimatized for a period of three days in the new environment before the initiation of experiment. The litter in the cages was renewed thrice a week to ensure hygiene and maximum comfort for animals. Ethical clearance for handling the animals was reviewed and approved by the University Animals Ethical Committee.

### Acute toxicity studies

Acute oral toxicity (ACT) of *S. Macrostema* was determined using Swiss albino mice. The animals were fasted for 12 h before the experiment and were administered with single dose of extracts dissolved in 5% gum acacia and observed for mortality up to 48 h (short term toxicity). On the basis of short-term toxicity, the dose of next animal was determined as per CECD guideline 420. The limit test carried out first at 4 g/kg. b.w. All animals were observed for toxic symptoms and mortality for 72 h.[7]

### Preparation of plant extracts

Hundred grams of the aerial parts were dried and powdered in a mechanical grinder. The powdered material was extracted by 500 ml of hexane, chloroform, methanol and water consecutively using a Soxhlet apparatus. These extracts were filtered and concentrated by a rotary vacuum evaporator and kept in a vacuum dessicator for the complete removal of solvent. Aqueous suspension of SM was prepared using 2% (v/v) Tween-80 and used for administration.

### Determination of total phenolic compounds in the extracts

The total phenolic content was determined using the Folin–Ciocalteu method.[8] The reaction mixture contained 1.0 ml of SM (10 mg), 10.0 ml of distilled water, and 1.5 ml of the Folin–Ciocalteu reagent. After a period of 5 min, 4.0 ml of 20% sodium carbonate solution was added and made up to 25 ml with distilled water. This mixture was shaken and allowed to stand for 30 min. The absorbance was measured at 765 nm. The percentage of total phenolic content was calculated from the calibration curve of gallic acid plotted and total phenolic content was expressed as mg GAE (gallic acid equivalents)/g dry extract.

### Determination of total flavonoid content in the extracts

The total flavonoid content was determined spectrophotometrically according to Lamaison and Camat.[9] The reaction mixture contained 0.5 ml of 2% aluminum chloride (AlCl_3_) ethanol and 0.5 ml of SM (1 mg/ml). Absorption readings at 415 nm were taken after 1 h against a blank (ethanol). The total flavonoid content was expressed as mg of quercetin equivalents/g of dry extract.

### Antioxidant activity *in vitro*

#### Inhibition of DPPH radical

The free radical scavenging activity of the extract was analyzed by the DPPH (1,1-- diphenyl-- 2--picryl hydrazyl) assay.[10] A total of 2 ml of the test extract, at concentrations ranging from 1 μg/ml to 100 μg/ml each, was mixed with 1 ml of 0.5 mM DPPH (in methanol). The absorbance at 517 nm was taken after 30 min of incubation in the dark at room temperature. The experiment was done in triplicate. The percentage antioxidant activity was calculated as follows:

%Antioxidant Activity [AA] = l00 --[ { (Abs_sample_ -- Abs_blank_) X 100 }/Abs ml of methanol plus 2.0 ml of the extract was used as the blank while 1.0 ml of the 0.3 mM

DPPH solution plus 2.0 ml of methanol was used as the negative control. Ascorbic acid was used as the reference standard.

#### Inhibition of superoxide anion radical

Measurement of superoxide anion scavenging activity of SM was performed based on the method described by Nishimiki[11] and slightly modified. About 1 ml of nitroblue tetrazolium (NBT) solution containing 156 μM NBT which is dissolved in 1.0 ml of phosphate buffer (100 mM, pH 7.4), 1 ml of NADH solution containing 468 μM of NADH which is dissolved in 1 ml of phosphate buffer (100 mM, pH 7.4) and 0.1 ml of various concentrations of SM and the reference compounds (5, 10, 25, 50 and 100 μg) were mixed and the reaction started by adding l00 μl of phenazine methosulphate (PMS) solution containing 60 μM of PMS l00 μl of phosphate buffer (100 mM, pH 7.4). The reaction mixture was incubated at 25°C for 5 min and the absorbance at 560 nm was measured against the control samples. All the tests were performed in triplicate and the results were averaged. The percentage decrease in absorbance was calculated.[10] Quercetine was used as the standard.

#### Inhibition of nitric oxide radical

Nitric oxide generated from sodium nitroprusside in aqueous solution at physiological pH interacts with oxygen to produce nitrite ions, which were measured by the Griess reaction.[12,13] The reaction mixture (3 ml) containing sodium nitroprusside (10 Mm) in phosphate buffered saline (PES) and SM and the reference compound in different concentrations (5, 10, 25, 50 and 100 μg) were incubated at 25°C for 150 min. In each 30 min, 0.5 ml of the incubated sample was removed and 0.5 ml of the Griess reagent (1% sulfanilamide, 0.1% naphthyl ethylene diamine dihydrochloride in 2% H_3_PO_4_) was added. The absorbance of the chromophore formed was measured at 546 nm. All the tests were performed in triplicate and the results were averaged. BHT was used as the reference compound. All the tests were performed in triplicate and the results were averaged. The percentage decrease in absorbance was calculated.[10] Quercetine was used as the standard.

#### Iron-chelating activity

Chelating of iron (II) ions by extracts was carried out as described in the previous work.[14] Briefly, a given volume of extracts (0.1222 mg/ml), ascorbic acid (0.1564 mg/ml), or BHT (0.1890 mg/ml) was added to 50 μl of 2.0 mM aqueous FeSO_4_ in a 5.0 ml test tube, then was added 1 ml of ethanol to complete 4,0 ml. After 5 min incubation, the reaction was initiated by 1.0 ml of 5.0 mM ferrozine. After 10 min of equilibrium, the absorbance at 562 nm was recorded. The controls contained all reaction reagents except extracts or positive control substance. Three experiments were performed and the average result was adopted. The iron-chelating activities were calculated from the absorbance of the control (Ac) and of the sample (As) using the following equation:

Inhibition(%) = Ac −As × 100 Ac

#### Hydroxyl radical scavenging assay

The OH· scavenging ability was evaluated as the inhibition rate of deoxyribose oxidation by this radical as described by Hutadilok--Towatana.[15] Tannic acid was used as the positive control. The capability to scavenge OH· was calculated based on the concentration of extract required to inhibit deoxyribose attack by 50% (IC_50_).

### Hepatoprotective activity

#### Induction of in vivo carbon tetra chloride hepatotoxicity

The animals were divided into control, carbontetrachloride (CCl_4_) and test groups (CCl_4_ + extracts, silymarin and extracts) each containing six animals in all the sets of experiments. 50% v/v CCl_4_ solution in olive oil was used for administration.[16] Animals from the control group received a single daily dose of 4% w/v aqueous tween-80 solution (1 ml/Kg i.p.) on all four days and olive oil (1 ml/kg s.c.) on day 2 and 3. Animals from CCl_4_ group received a single daily dose of 4% w/v aqueous tween-80 solution (1 ml/kg i.p.) for four days and CCl_4_ solution 2 ml/kg s.c. on day 2 and 3, 30 min after the administration of aqueous tween-80 solution. Animals from the test groups received single daily dose of the extracts (200, 400 y 600 mg/kg mg/kg i.p.) and silymarin (50 mg/kg i.p) for four days. The animals were also administered toxicant CCl_4_ (2 ml/kg s.c) 30 min after the administration of the test extracts.

#### Induction of in vivo paracetamol hepatotoxicity

The animals were divided into control, paracetamol (Pcl) and test groups (Pcl + extracts and silymarin) each containing six animals in all the sets of experiments. Pcl was suspended in 60% w/v aqueous sucrose solution. Animals from the control group received a single daily dose of 4% w/v aqueous acacia solution (1ml/Kg i.p.) on all three days and a single dose of 60% w/v sucrose solution (1 ml/kg p.o.) on day 3. Animals from Pcl group received a single daily dose of 4% w/v aqueous acacia solution (1 ml/kg i.p.) for three days and single dose of Pcl suspension 3 g/kg p.o. on day 3, 60 min after the administration of aqueous tween-80 solution. Animals from the test groups received a single daily dose of the extracts (200, 400 y 600 mg/kg mg/kg i.p.), silymarin (50 mg/kg i.p) for three days. The animals were also administered toxicant single dose of Pcl suspension (3 g/kg p.o.) on day three, 60 min after the administration of the test extracts.[17]

#### Biochemical studies

At the end of the experimental period (CCl_4_ hepatotoxicity), animals were sacrificed by cervical decapitation, blood was collected and serum was separated for biochemical markers of liver damage like serum glutamate pyruvate transaminase (SGPT),[18] serum glutamate oxalaoacetate transaminase (SGOT),[19] alkaline phosphatase (ALP),[20] serum bilirubin,[21] cholesterol[22] and HDL;[23] alanine aminotransferase (ALT) and aspartate aminotransferase (AST) were assayed in serum by the colorimetric test of Rodier and Mallein[24] as toxicity marker enzymes.

The hepatoprotective activity was calculated as:

[1-- (ALTdrug --ALTcontrol/ALT CCl_4_ --ALTcontrol)] X 100

In the case of induction of *in vivo* paracetamol hepatotoxicity, the activity was assessed by estimating serum transaminases viz. glutamyl pyruvate transaminase (GPT) and glutamyl oxalacetate transaminases (GOT) according to the method of Reitman and Frankel.[25] Also biochemical markers of live damage alkaline phosphatase (ALKP)[22] and total bilirubin (TB)[26] were determined.

#### Statistical analysis

Results of biochemical analysis are presented as mean values± S.D. and % reduction was calculated by considering the difference between the control and the toxicant as 100% reduction. Statistical significance of the difference was analyzed through one way analysis of variance (ANOVA) by SPSS version 11.5 for Windows. Difference between the test group and the control was determined by least significant difference method at *p*<0.05 confidence levels.

## RESULTS

### Acute toxicity studies

For acute oral toxicity studies, the extract-treated animals were observed for mortality upto 72 h. On the basis of the results, it can be seen that the extract did not produce any mortality up to 4000 mg/kg body weight.

### Total phenolic and flavonoid content of the methanol extract

The percentage of total phenolics in the SM was expressed as 172.53 ± 6.2 mg gallic acid equivalents (GA)/g dry weight of extract. Along with this, total flavonoid content was also measured and it was 78 ± 4.8 mg quercetin equivalents (QE)/g dry weight of extract.

*In vitro* antioxidant activity and DPPH radical scavenging activity of the methanol extracts

DPPH radical scavenging activity of the methanol extracts of the leaves of *S. macrostema* was compared with those of ascorbic acid and quercetin. The DPPH radical scavenging abilities of the extracts (89.87%) were found to be less than those of ascorbic acid (97%)) at 100 μg/ml [Table 1].

**Table 1:** *In vitro* antioxidant effect of methanol extract of *S. Macrostema* (SM)

Treatment mg/ml	DPPH scavenging	Nitric oxide scavenging	Superoxide anion scavenging
SM 20	27.45 ± 0.34*	19.34 ± 1.23*	20.98 ± 0.13*
SM 40	38.16 ± 0.23*	28.34 ± 2.56*	36.45 ± 0.17*
SM 60	53.27 ± 0.19*	42.57 ± 3.15*	48.79 ± 0.20*
SM 80	68.98 ± 0.42*	59.76 ± 1.98*	54.36 ± 0.24*
SM 100	89.87 ± 0.39*	69.79 ± 4.17*	66.12 ± 0.25*
Ascorbic acid 100 (μg/ml)	97 ± 0.52*	-	-
Quercetin 50 (μg/ml)	-	93.8 ± 0.98*	90.87 ± 0.43*

Effect of different concentrations of SM, ascorbic acid and quercetin on DPPH free radical, nitric oxide and superoxide anion scavenging activities.

* Data are mean representative of three experiments and the result are expressed as Mean ± S.E.M.

Inhibition of nitric oxide scavenging and superoxide anion scavenging of the methanol extracts

Table 1 shows the dose--response results of nitric oxide scavenging and superoxide anion scavenging of the methanol extracts of the leaves of *S. macrostema*. The extract reduced the generation of nitric oxide radicals from sodium nitoprusside solution. This showed marked nitric oxide scavenging of the extract (69.79%). Also the extract showed significant superoxide scavenging activity (76.12 %) at 100 μg/ml.

### Iron-chelating activity

In this study, the chelations of ferrous ions by extracts, ascorbic acid and BHT as controls were estimated. In the presence of chelating agents, the complex formation is disrupted, resulting in a decrease in the red color. The efficiencies of Fe^2+^--ferrozine complex increase with the increasing concentration of the three antioxidants. In this assay, methanol extract showed iron-chelating activity [Table 2]. The median inhibitory concentration (IC_50_) values for methanol extract, ascorbic acid and BHT were 66.5, 49.3 and 46.6 μg /ml, respectively.

**Table 2:** Ion-chelating and OH. scavenging activities of methanol extract of *S. Macrostema* (SM)

Treatment	IC50 (mg/ml)	
	
	Ion-chelating	OH scavenging
SM	66.5	49.65
Ascorbic acid	49.3	-
BHT	46.6	-

### Hydroxyl radical scavenging assay

In Table 2, methanol extract shows highest activity on OH· scavenging with IC_50_ values of 49.65 μg/ml.

### Hepatoprotective effect

In addition to antioxidant, the ability of hepatoprotective action of SM was assessed by measuring the level of biochemical enzyme. As shown in Table 3, administration of CCl_4_ significantly enhanced the biochemical markers like ALT, AST, SGPT, SGOT by three to four fold. ALP, total bilirubin, cholesterol and reduced levels of HDL are shown in Table 4. Pretreatment with SM (200, 400 and 600 mg/kg) reduced the elevated levels of all the above-mentioned biochemical indicators and increased the level of HDL. The groups treated with the hexane and chloroform extracts did not reduce the elevated biochemical parameters, indicating no protection

**Table 3:** Effect of different doses of methanol extract from *S. Macrostema* (SM) on ALT, AST, SGPT and SGOT on CCl_4_-induced hepatotoxicity in rats

Group (mg/kg)	ALT (IU/L)	AST (IU/L)	SGPT (U/I)	SGOT (U/I)
Control		61.21 ± 1.43	48.12 ± 1.86	100.98 ± 0.45
CCl_4_		141.89 ± 1.96**	295.11 ± 1.72**	421.41 ± 0.15**
SM 200		109.69 ± 1.65*	145.70 ± 1.95*	249.76 ± 0.34*
SM 400		89.21 ± 2.75*	103.05 ± 1.96*	166.59 ± 0.23*
SM 600		73.43 ± 0.97*	78.76 ± 2.34*	140.98 ± 0.19*
Sylimarin 100		105.61 ± 1.47*	62.75 ± 2.06*	132.62 ± 0.43*

Each value represents the mean ± SEM, n = 5;

* *P*<0.05 significantly different values from CCl_4_ group.

** *P*<0.01 indicate significantly values compared to control group.

**Table 4:** Effects of different doses of methanol extract from *S. Macrostema* (SM) on ALP, total bilirubin cholesterol and HDL on CCl_4_-induced hepatotoxicity in rats

Group (mg/kg)	ALP (U/I)	Total bilirubin (mg/dl)	Cholesterol (mg/dl)	HDL (mg/dl)
Control	135.67 ± 0.07	0.98 ± 2.45	105.47 ± 3.24	47.89 ± 1.29
CCl_4_	257.38 ± 0.09**	4.56 ± 2.03**	175.36 ± 2.74**	27.91 ± 1.56**
SM 200	148.90 ± 0.06*	2.64 ± 2.87*	141.32 ± 4.32*	34.12 ± 1.70*
SM 400	119.80 ± 0.04*	1.89 ± 1.99*	136.73 ± 4.56*	38.61 ± 1.86*
SM 600	95.99 ± 0.02*	1.25 ± 2.65*	124.50 ± 3.75*	46.02 ± 1.73*
Sylimarin 100	96.45 ± 0.03*	1.28 ± 2.84*	122.61 ± 3.56*	47.54 ± 1.28*

Each value represents the mean ± SEM, n = 5;

* *P*<0.05 significantly different values from CCl_4_ group.

** *P*<0.01 indicate significantly values compared to control group

Administration of paracetamol resulted in elevated levels of GPT, GOT, AKKP and TB by three to four folds indicating development of hepatotoxicity. Administration of the different doses of methanol extract caused significant reduction in biochemical parameters, whereas the groups treated with the hexane and chloroform extracts could not reduce the elevated biochemical parameters indicating no protection. Pretreatment with the methanol extract significantly reduced GPT, GOT and ALKP levels as compared to paracetamol [Table 5].

**Table 5:** Effects of methanol extract from *S. Macrostema* (SM) on biochemical parameters in rats intoxicated with paracetamol

Group (mg/kg)	% Reduction			
	
	GPT (U/L)	GOT (U/L)	ALKP (U/L)	TB (U/L)
Control	69.21 ± 3.45	107.45 ± 3.47	47.22 ± 2.34	0.24 ± 1.67
Paracetamol	126.65 ± 2.96**	244.53 ± 2.64**	175.21± 6.21**	1.73 ± 1.98**
SM 200	112.65 ± 4.03*	199.94 ± 1.76*	151.01 ± 2.23*	1.54 ± 0.54*
SM 400	81.02 ± 3.65*	166.47 ± 1.57*	135.72 ± 3.42*	1.05 ± 0.87*
SM 600	70.36 ± 2.43*	141.30 ± 1.83*	109.17 ± 2.56*	0.69 ± 0.69*
Sylimarin 50	66.23 ± 2.54*	139.92 ± 1.25*	105.34 ± 2.19*	0.60 ± 0.43*

Each value represents the mean ± SEM, n = 5;

* *P*<0.05 significantly different values from paracetamol group.

** *P*<0.01 indicate significantly values compared to control group

## DISCUSSION

It has been already reported that phenolic compounds play an important role in scavenging of free radicals. The correlation between antioxidant activities and quantity of the flavonoids is still under discussion, a good linear relationship was observed in some published works.[27,28] However, Hinneburg[29] found no linear relationship between them. The controversy might be contributed to the complexity of plant materials used by them. Results obtained in the present study revealed that the level of these phenolic compounds in the methanol extracts of the leaves and stem of *S macrostema* was considerable. Polyphenolic compounds are known to have antioxidant activity and it is due to their redox properties, which play an important role in adsorbing and neutralizing free radicals, quenching singlet and triplet oxygen, or decomposing peroxides.[30,31] In fact, many medicinal plants contain large amounts of antioxidants such as polyphenols, many of these phytochemicals possess significant antioxidant capacities that are associated with lower occurrence and lower mortality rates of several human diseases. The results strongly suggest that phenolics are important components of this plant, and some of its pharmacological effects could be attributed to the presence of these valuable constituents.[32] The effect of antioxidants on DPPH is considered to be due to their hydrogen donating ability.[33] The present study shows that the extracts have the proton-donating ability and could serve as free radical inhibitors or scavengers, acting possibly as primary antioxidants.

The methanol extract of SM shows significant superoxide anion, nitric oxide scavenging activities in a dose dependent manner. Simultaneous generation of NO and O_2_^--^ favors the production of a toxic reaction product, peroxynitrite (ONOO^--^). The scavenging of the superoxide anion and nitric oxide indicate the possibility of preventing the formation of peroxynitrite in the cell. Reducing the nitric oxide generation in the digestive tract was found to be effective in preventing the reactions of nitrate with amines and amides to form carcinogenic nitrosamines and nitrosamides.[34] Hence the NO scavenging activity of SM extract could play a preventive role against nitrosamine-mediated carcinogenesis.

Iron-chelating capacity is important as it reduces the concentration of the catalyzing transition metal in lipid peroxidation via the fenton reaction. Ferrozine can quantitatively form complexes with Fe^2+^chelating agents, which form δ-bonds with a metal, that are effective as secondary antioxidants because they reduce the redox potential and then stabilize the oxidized form of the metal ion.[35]

OH· scavenging activities were determined based on the ability of the antioxidant components in the samples to inhibit deoxyribose oxidation by reactive OH· generated from Fenton's type reaction.[36] In this case, two anti oxidation mechanisms are involved. One is the suppression of the OH· generation from H_2_O_2_ by binding with metal ions and the other is a direct single electron transfer to the generated radical. *S. Macrostema* is high in polyphenols that are known to be strong chelators of heavy metals, and are also believed to be related to such effective OH· scavenging ability. Apart from the phenolic compounds that are responsible for the antioxidant activity, there might be some other active compounds that also exert some effects.

This present study evaluated the hepatoprotective activities of *S. Macrostema* in CCl_4-_ and paracetamol-induced liver toxicity. It is generally accepted that the hepatotoxicity of CCl_4_ depends on the cleavage of the carbon--chlorine bond to generate tricloromethyl free radical (.CCl_3_); this free radical reacts rapidly with oxygen to form a trichloromethyl peroxy radical (.CCl_3_O_2_). This metabolite may attack membrane polyunsaturated fatty acids and causes lipid peroxidation which plays a main role in the induction of liver injury[37] and further causes impairment of membrane function. CCl_4_-induced hepatic injuries are commonly used as models for the screening of hepatoprotective plant extract and the extent of hepatic damage is assessed by the level of released cytosolic transaminases including ALT and AST in circulation.[38] When administrated prophylacticaly, methanol extract exhibited protection against CCl_4_-induced liver injuries as manifested by the reduction of toxin-mediated rise in serum enzymes in rats.

Paracetamol is a common antipyretic agent, which is safe in therapeutic doses but can produce fatal hepatic necrosis in man, rats and mice with toxic doses.[39] Protection against paracetamol-induced toxicity has been used as a test for potential hepatoprotective activity by several investigations. The covalent binding of N--acetyl--*p*--benzoquinoneimine, an oxidation product of paracetamol, to sulfhydryl groups of protein resulting in cell necrosis and lipid peroxidation induced by a decrease in glutathione in the liver as the cause of hepatotoxicity has been reported earlier.[40] This is one of the most important natural antioxidants of the hepatocytes that renders the cell remarkably susceptible to oxidative stress.

In the assessment of liver damage by paracetamol, enzyme levels such as GOT and GPT are mainly determined. Necrosis or membrane damage releases the enzyme in to circulation; therefore, it can be measured in serum. A high level of GOT indicates liver damage such as that due to viral hepatitis as well as cardiac infarction and muscle injury. GPT catalyzes the conversion of alanine to pyruvate and glutamate, and is released in a similar manner. Therefore, GPT is more specific to the liver, and is thus a better parameter for detecting liver injury.[41] Elevated levels of serum enzymes are indicative of cellular leakage and loss of functional integrity of cell membrane in liver. Prolonged destruction of the hepatic cells results in more hepatic releases to exacerbate hepatic dysfunction and causes an elevation in the serum levels of ALP, LDH, and bilirubin.[42]

Acute administration of paracetamol produced a marked elevation of the serum levels of GOT, GPT, ALKP serum bilirubin in treated animals when compared with that of control group. Treatment with methanol extract significantly reduced the elevated levels of the enzymes toward the respective normal value that is an indication of stabilization of plasma membrane as well as repair of hepatic tissue damage caused by paracetamol. The above changes can be considered as an expression of the functional improvement of hepatocytes, which may be caused by an accelerated regeneration of parenchyma cells. Effective control of alkaline phosphatase (ALKP) and bilirubin levels points toward an early improvement in the secretary mechanism of the hepatic cell.

The antioxidant activity may be due to the inhibition of the formation of radicals or scavenging of the formed radical and the presence of the phenolic compounds. These results concluded that SM has promising antioxidant and hepatoprotective effects. The findings thus establish the potential medicinal value of the plant *S. Macrostema* used in indigenous systems of medicines in Mexico and also initiate further detailed investigations on this plant in order to justify its use in polyherbal formulations prescribed in the treatment of liver disorders.
